# Temporal Course of Interference Control from Early to Late Young Adulthood: An ERP Study

**DOI:** 10.3390/brainsci14060536

**Published:** 2024-05-24

**Authors:** Martina Knežević

**Affiliations:** Department of Psychology, Catholic University of Croatia, Ilica 242, 10000 Zagreb, Croatia; martina.knezevic@unicath.hr

**Keywords:** interference control, young adulthood, ERP, Stroop task, protracted development, early 20s

## Abstract

In the present study, we aimed to investigate the neural dynamics of interference control using event-related potentials (ERPs) to reveal time course of interference control from the beginning to the end of young adulthood. Three groups of participants aged 19–21, 23–27 and 28–44 performed a Stroop task. The results revealed age differences in both accuracy and ERP amplitudes during all aspects of interreference control processing that reflect selective attention (P2), conflict monitoring (N2), conflict evaluation (P3) and interference control (N450). Both younger groups made more errors on incongruent trials compared to participants in their early 30s. The presence of higher P2 and N2 amplitudes, diminished P3 and again higher N450 amplitudes in participants in their early 20s points to a shortage of available resources for top-down control at this age. These results are in accordance with structural and functional studies that show that development of the frontoparietal network, which underlies interference control, continues after adolescence. While brain mechanisms are still developing, the use of accompanying cognitive abilities is still not optimal. The findings that change in neural dynamics and related performance continues into early adulthood challenge current models of cognitive development and call for new directions in developmental theorizing.

## 1. Introduction

After adolescence, a young individual suddenly enters the world of adults with somewhat different responsibilities and expectations, such as finding full-time employment and thus achieving financial independence, as well as facing challenges of independent living and forming a family. These life-changing circumstances require particular skills, such as the capacity to plan ahead, make appropriate decisions, display self-control and stay focused, which are essential for success in everyday living [1]. This set of interconnected cognitive abilities, which are vital for self-monitoring and changing behavior flexibly and in accordance with individual goals and current circumstances, is known under an umbrella term—executive functions [2]. Executive functions show protracted development from childhood into early young adulthood, and this has been associated with structural and functional brain maturation [3,4]. Many studies have so far found that brain maturation continues until around 28 years of age [5,6]. One of the main problems with current evidence about developmental trajectories of executive functions lies in the selection of age groups. Whereas cellular, structural and functional studies in the last 20 years argued that brain restructuration continues well into the early 20s, the majority of cognitive development studies compare dichotomous adolescent and adult age groups collapsing across 18- to 30-year-olds.

To investigate if and how the experience of transitioning to adulthood and the protracted brain development foster cognitive development, in several of our previous studies we focused on early adult years [7,8,9,10], dividing young adults into those in their early and mid 20s and those above 28, the age after which significant maturational changes are not expected anymore [6]. We first explored response inhibition as one of the key executive functions for voluntary control of behavior, using event-related potentials (ERPs) to accurately trace the temporal flow of response inhibition components in the brain [8]. We found that response inhibition was not at the adult level at the beginning of young adulthood, since participants in their early 20s showed more premature, impulsive responses coupled with differences in P2-N2-P3 ERP components which reflect attentional control (P2), performance optimization (N2) and response activation or inhibition (P3) in response inhibition tasks, compared to participants in their early 30s. Error analysis [9] showed further differences between these age groups. After the impulsive errors, contrary to young adults in their early 30s, those in their early 20s did not show post-error adjustment (PES) important for performance improvement, which was reflected in age differences in brain indicators of error detection (error-related negativity or ERN) and error awareness (error positivity or Pe). Several other studies also investigated the development of inhibitory control during and after adolescence. Using functional magnetic resonance imaging and a response inhibition task, Rubia et al. [11] investigated differences in brain activation between a group of 10–17-year-old adolescents and a group of 20–43-year-old adults. They found increased brain activation in adults compared to adolescents in task-specific fronto-striatal networks during response inhibition—that is, the right orbital and medial prefrontal cortex. Knežević et al. [7] found more activation in the right inferior frontal gyrus during the performance monitoring portion of the response inhibition task in young adults aged 18–19 compared to young adults aged 23–25. Theodoraki et al. [12] found developmental changes in performance on the response inhibition task in a cross-sectional sample of adolescents aged between 14 and 18 years, while Ferguson et al. [13] found maturational changes throughout adolescence (aged 10–17 years) and young adulthood (aged 18–29 years) in response inhibition, indicating its protracted development into early adulthood.

We also investigated the efficiency of applying cognitive control using a cognitive task in which participants were asked to classify different words into categories (perceptual and semantic). During the task we recorded EEG responses [10]. Our study revealed differences in performance and ERP amplitudes: participants in their early 20s made more performance errors during the task, while enlarged P2 and N2 ERP amplitudes indicated that they had to apply more cognitive focus and attention to resolve the task, contrary to young adults in their early 30s. Since previous studies provided proof of continuing age-related changes in neural corelates that underlie response inhibition and cognitive efficiency during the early 20s, here we decided to investigate one more important aspect of executive functioning—interference control. Interference control refers to the ability to suppress stimuli that are interfering with current working memory operations important to carrying out correct motor response [14]. Indeed, successful performance often depends on the ability to focus resources on goal-relevant information, while filtering out or inhibiting competing but irrelevant information. One of the classic and widely applied cognitive interference tasks is the Stroop color–word task [15]. In such a task, color naming is faster when the color of the presented word and its meaning match (they are congruent, e.g., the word “red” printed in red color) relative to when they do not match (they are incongruent, e.g., the word “red” printed in blue color). Improvements in interference control have been found from middle childhood to late adolescence and early adulthood [14,15]. Neuroimaging studies have shown consistent involvement of the anterior cingulate cortex, along with the dorsolateral frontal cortex, the orbital frontal cortex, the superior and inferior parietal lobe, and the insular cortex in interference control [16,17]. While neuroimaging methods can identify brain areas that are engaged in different tasks [18,19], scalp ERPs with their excellent temporal resolution measure processing in a continuous manner, and as such provide precise detection of stages between a stimulus and a response [20]. Therefore, ERPs are particularly important for interpretation of the course of development for complex cognitive processes, such as executive functions.

Several ERP components related to the color–word Stroop task have been researched extensively. The P2 is a positive potential that peaks around 200 ms after stimulus presentation at fronto-central electrodes. This component is sensitive to stimulus features, such as color, and is usually interpreted as indexing early attentional processing [21]. When conflict between two responses arises, the ERPs show a negative N2 deflection peaking between 250 ms and 350 ms over the fronto-central scalp regions [22]. Using a dipole source analysis, previous studies suggested that the N2 is generated in the anterior cingulate cortex (ACC), which shows greater activity as the conflict is higher [23]. Next comes the P3 component most prominently over the fronto-central scalp regions between 350 ms and 450 ms after stimulus onset, reflecting stimulus evaluation and categorization, and its source is estimated to be the inferior prefrontal cortex [24,25]. The N450 emerges immediately after the P3 as a reduced positive component (negativity) over the frontal-central region between 450 and 550 ms after the stimulus onset [23,26]. The neural generator of the N450 has been estimated to be the ACC as well, and this component is suggested to reflect conflict resolution [27].

In the present study, our aim was to investigate age-related differences in interference control from early to late young adulthood by applying a modified Stroop task during which an EEG signal was recorded. The modified Stroop task included three conditions: congruent, in which the color of the word and its meaning matched, incongruent, in which the color of the word and its meaning conflicted, and read, which included color words written in gray. In the first two conditions participants responded to the color of the word, while in the last (read) condition participants responded to the meaning of the word. The read condition was introduced to support reading dominance and automaticity. At the beginning of young adulthood, individuals are faced with numerous challenges which they have to overcome to become functioning adults. Success or failure at this stage may set the course that will greatly influence their adult lives. In addition, detecting patterns of typical development after adolescence is very important because mental disorders at this age are frequent and generally co-morbid [28,29]. For example, schizophrenia and psychotic symptoms (such as hallucinations and delusions) usually show up in the late teens and early 20s [30], as well as generalized anxiety disorder or depression, which are most common between the ages of 18 and 25 [29]. As such, the early 20s can be considered a vulnerable period for psychopathology. Many of these disorders are characterized by impairments in interference control [31]. Based on our previous results [7,8,9,10], we hypothesized that interference control would continue to develop throughout the early 20s, which will be shown by improvements in performance and differences in ERP components from the early 20s to the early 30s.

## 2. Materials and Methods

### 2.1. Participants

We included 131 volunteers in this study and divided them into age groups based on the results from our previous studies [7,8,9,10]: Early 20s (*n* = 44; 22 females; *M* = 19.8; *SD* = 0.78; age range 19–21 years), Mid 20s (*n* = 44; 23 females; *M* = 24.5; *SD* = 1.02; age range 23–27 years) and Early 30s (*n* = 43; 21 females; *M* = 33.3; *SD* = 4.24; age range 28–44 years). An additional three participants were excluded from the analysis because they had more than 20% of blink artifacts and artifacts due to involuntary muscle contractions.

The participants provided responses about possible EEG contraindications, such as previous diagnoses, head injuries or various prescription drugs. They did not report any history of mental illness, previous head injuries or using any medication at the time of this study. All were right-handed [32] and their vision was either normal or corrected-to-normal. None of them reported any reading difficulty and all were native Croatian speakers.

The participants were recruited at the University of Zagreb where we posted advertisements, as well as through social networking. Each was briefed before the onset of the experiment, with additional feedback at the end. This study was conducted in line with the ethical standards of the American Psychological Association (APA) and the 1964 Declaration of Helsinki. The local University of Zagreb’s Ethics Committee approved this study (Class: 643-02/09-03/22, Reference number: 380-02/7-11-6, 30 November 2011).

Participants gave written informed consent and were given detailed information about their right to withdraw from the research at any time. In case of any additional questions or concerns, they were provided with the principal investigator’s phone number and e-mail.

### 2.2. Psychological Testing Battery

To assess personality traits, we used the Barratt Impulsiveness Scale (BIS) [33] and the Eysenck Personality Questionnaire (EPQ) [34]. BIS is a measure of impulsivity. It contains 15 items that are self-rated on a Likert-type scale (1 = rarely/never, 4 = almost always). Summed scores represent the level of impulsivity. EPQ assesses extraversion, neuroticism and psychoticism with 90 yes or no items, and the result is calculated for each trait separately as the sum of scores of corresponding items.

To estimate a non-verbal g-factor IQ, we used the Cognitive Nonverbal Test [35] which comprises 40 items, each containing four geometrical shapes. Within a 15 min time limit, participants were required to mark the one shape that differs from the other three.

To measure information processing speed, we used the Letter Digit Substitution Test (LDST) [36]. Participants’ task was to, as quickly and accurately as possible, replace the randomized letters with the correct number indicated by the key. The key contained numbers ranging from 1 to 9, and each number was paired with a different letter from the alphabet. The final outcome is calculated as the sum of correct substitutions within a 60 s time limit.

### 2.3. The Task

**Figure 1:**
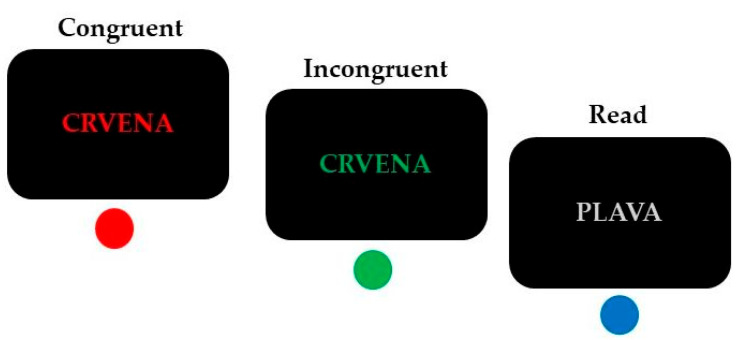
Trial examples of the conditions (incongruent, congruent, and read) in the modified four-color Stroop task (“crvena” = red; “plava” = blue). Correct responses are indicated below each example.

A modified four-color Stroop paradigm [37] was used to investigate interference control (Figure 1).

This task combines reading and color-naming trials in a randomized manner. The stimuli were the Croatian words “plava” (blue), “zelena” (green), “crvena” (red) and “žuta” (yellow), presented on a black background (font: Arial, 30 pt). Response conflict was elicited during the incongruent condition when the color of the displayed word and its meaning did not match (e.g., the word ‘‘zelena” (green) printed in blue color), in comparison with congruent trials during which the meaning and the color of the displayed word corresponded (e.g., the word “zelena” (green) printed in green color). Participants were instructed to respond to the font color of these words.

An additional condition was added to maintain dominance and automaticity of reading [38]. This read condition included four color words (“crvena”, “žuta”, “zelena” and “plava”) written in gray, and participants’ task was to press the button that corresponded to the meaning of the word. This control condition was not further analyzed since such a task should be relatively effortless to resolve for skilled readers.

E-prime 2.0 (Psychology Software Tools, Pittsburgh, PA, USA) was used for task programing, along with Serial Response Box (S-R Box) for response collection due to its 0 millisecond debounce period. Participants responded using their index fingers for yellow (left) and green (right), and middle fingers for red (left) and blue (right). Each stimulus was displayed for 500 ms, with 1500 ms ISI. A total of 400 stimuli were presented, including 100 items in congruent condition, 100 items in incongruent condition and 200 items in read condition, separated into 3 blocks and counterbalanced across participants.

### 2.4. Procedure

**Figure 2:**
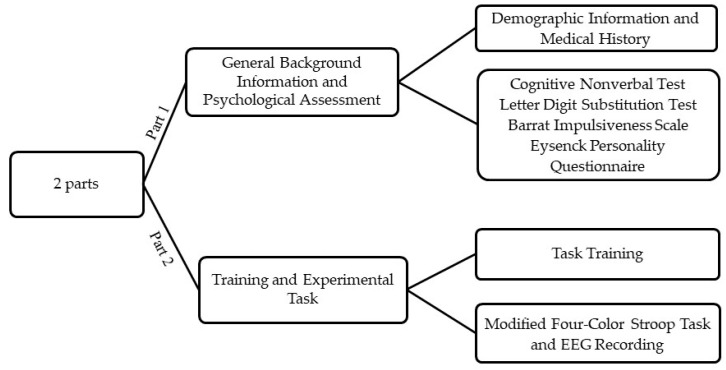
A schematic workflow of the study procedure.

This study was carried out at the University of Zagreb, Croatia in the Laboratory for Psycholinguistic Research. Participants were first given a detailed description of the procedure, laboratory and the task in order to minimize the potential effects of situation-induced arousal. All gave written informed consent and thereafter provided demographic information, medical history, and completed the psychological testing battery (Figure 2).

Each participant received a short training to ensure correct performance. They first practiced responding to strings of X’s written in color (red, yellow, green and blue), followed by responding to color words written in gray, and then a combination of X’s written in color and color words written in gray. After successful response mapping, they were given a block of practice trials with congruent, incongruent and read stimuli.

After the training, participants were seated in an office chair at a normal viewing distance at approximately 80 cm relative to a 24′′ computer monitor (Samsung SyncMaster T220) within a sound-attenuated room. A 32-channel EEG cap was fitted (actiCAP, Brain Products GmbH, Munich, Germany), along with two vertical and two horizontal electro-oculogram (EOG) electrodes for eye saccades and blinks recording. Each block was preceded by instructions on the screen and all participants were reminded to be accurate and fast.

### 2.5. Data Recording and Analysis

We used Brain Vision (version 1.03) recording system (Brain Products GmbH, Gilching, Germany) with a standard 32-channel actiCAP EEG cap, 0.01–100 Hz band pass and 1 kHz sampling rate. Active electrodes were used at all sites except vertical and horizontal EOG, which were Ag/AgCl sintered electrodes. Recordings started when electrical impendence was below 5 K-Ohm. During the recording, we used FCz electrodes as a reference. Before further data processing, we used the average of right and left mastoids (TP9/TP10) to re-reference all electrodes.

For EEG data analysis, we utilized version 2.0 of the BrainProducts Analyzer software package (Brain Products GmbH, Gilching, Germany). EEG data processing was performed in line with our previously published studies [7,8,9,10], as well as in accordance with guidelines in the literature [20,39]. First, a band-pass filter from 0.1 Hz (12 dB/octave) to 30 Hz (12 dB/octave) was used to filter the continuous EEG recordings offline. Next, a computerized artifact rejection was performed in order to discard epochs with excessive eye movements, blinks, muscle potentials or amplifier blocking. To remove vertical and horizontal EOG that are embedded in the data, we utilized Independent Component Analysis (ICA) in a semiautomatic manner. During the averaging procedure, all sweeps in which the absolute difference between two adjacent data points exceeded 75 µV/ms, and the amplitude voltage exceeded ±100 µV at any scalp electrode, were edited out [39]. During the final preprocessing stage, the experimenter additionally checked all data.

All sweeps were aligned to a baseline from −200 to 0 ms preceding the stimulus. Stimulus-locked data were segmented into epochs ranging from 200 ms before to 1800 ms after the stimulus onset. Only trials with correct responses to congruent and incongruent stimuli were included in the analysis. The remaining epochs were averaged for each participant, and the proportion of rejected epochs varied between 6 and 9% per participant mainly due to blinks. Artifact-free, averaged ERPs were obtained for 83 (±13) trials in the incongruent condition and for 86 (±11) trials in the congruent condition. The components of interest were determined based on the inspection of the grand average waveforms for each age group and compared to the literature [40,41]. Next, mean amplitudes were computed for P2 from 150–250 ms time window, N2 over 250–350 ms interval, P3 over 350–450 ms interval and N450 over 450–550 ms interval for each participant and condition.

### 2.6. Statistical Analyses

SPSS version 23 (IBM SPSS Statistics for Windows, Armonk, NY, USA) was used for statistical analyses. The Kolmogorov–Smirnov test was used to check for normality, while Levene’s test of equality of error variances was used to test homogeneity of variances, as was Mauchly’s test of sphericity. The results showed that all variables were normally distributed. Based on sphericity and homogeneity of variance information, data transformation was not considered necessary. Univariate ANOVAs were conducted for the psychological testing battery, performance accuracy, reaction times and ERP components (P2, N2, P3, N450) for each condition (incongruent, congruent) independently. Age groups—Early 20s, Mid 20s and Early 30s—were used as the between-subjects factor. We report data for the frontal and central midline (Fz and Cz) electrodes where the components showed maximum amplitude and were in line with the available literature [12,15,40,41].

Group differences were checked using Tukey’s post-hoc test. We calculated partial eta squared (η_p_^2^) to show effect sizes, with 0.01–0.05 for small effect size, 0.06–0.13 medium effect size and 0.14+ large effect size. The standard level of significance *p* < 0.05 was adopted.

## 3. Results

### 3.1. Psychological Testing Battery

We did not find significant differences (Table 1) between age groups in personality traits, non-verbal IQ or speed of information processing, indicating that participants were well matched in these psychological characteristics.

### 3.2. Performance

**Figure 3:**
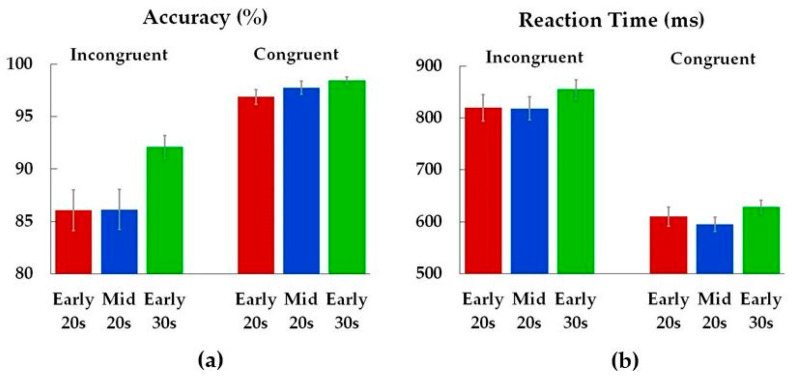
Accuracy (**a**) and reaction times (**b**) for incongruent and congruent conditions. Color bars represent means (*M*) for each age group (Early 20s, Mid 20s, Early 30s), while the error bars show standard errors (*SE*) of the means.

Analysis of performance accuracy revealed age differences on incongruent trials (Figure 3, Table 2). Early 30s were more accurate compared to both Early 20s (*p* = 0.04) and Mid 20s (*p* = 0.04). There was no difference in accuracy between Early 20s and Mid 20s (*p* = 1.00), and age groups did not differ in reaction times.

### 3.3. ERP Analysis

**Figure 4:**
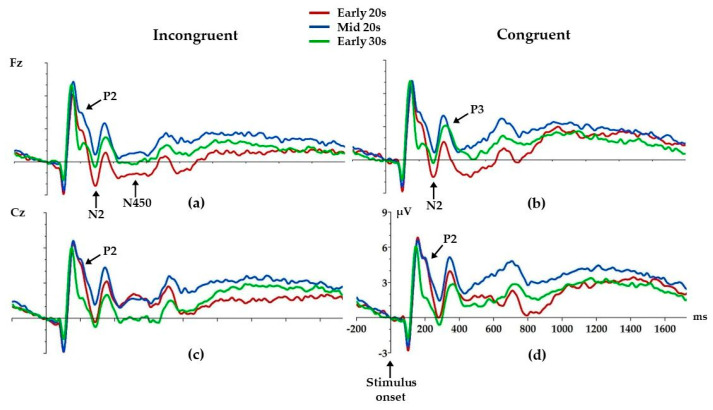
Grand average ERPs for each electrode and condition: Fz incongruent (**a**) and congruent (**b**), Cz incongruent (**c**) and congruent (**d**). Arrows mark ERP components where age differences were found. Positive is up.

We found age differences in P2 amplitudes at both the Fz and Cz electrodes (Figure 4, Table 3). Mid 20s had higher incongruent P2 amplitude compared to Early 30s (*p* = 0.01) at the Fz electrode. Figure 4 shows that there are similar age differences in P2 amplitudes in congruent condition; however, this difference did not reach significance. At the Cz electrode, age differences were found for both incongruent and congruent P2 amplitudes (Figure 4, Table 3). Both Early 20s (*p* = 0.02) and Mid 20s (*p* = 0.01) had higher incongruent P2 compared to Early 30s. Similarly, both Early 20s (*p* = 0.01) and Mid 20s (*p* = 0.01) had higher congruent P2 compared to Early 30s.

At the Fz electrode, age differences were found for incongruent and congruent N2, where Early 20s had higher incongruent (*p* = 0.01) and congruent (*p* = 0.02) N2 compared to Mid 20s. Similar differences can be observed at the Cz electrode (Figure 4); however, these differences did not reach statistical significance.

Early 20s had lower congruent P3 amplitude compared to both Mid 20s (*p* = 0.04) and Early 30s (*p* = 0.01) at the Fz electrode.

Early 20s also had higher incongruent N450 compared to Mid 20s (*p* = 0.003) and Early 30s (*p* = 0.02) at the Fz electrode. Similar effects can be observed at the Cz electrode; however, these differences did not reach statistical significance.

There were no other significant age differences.

## 4. Discussion

In the current study, we investigated the neural underpinnings of interference control using a modified version of the color–word Stroop task in order to reveal time-course of interference control development from the beginning to the end of young adulthood. Based on structural and functional brain development studies and our previous findings, we divided young adults into three age groups—Early 20s, Mid 20s and Early 30s—and found differences in performance and neurophysiological correlates (P2, N2, P3 and N450) of interference control between these groups.

Early 30s were more accurate on the incongruent trials compared to both Early 20s and Mid 20s. Studies show that reading automaticity is fully matured around age 12 [42]. One explanation is that interference on the Stroop task is a result of semantic conflict between color and meaning. More specifically, during the incongruent trials conflict arises between automatic language processing of reading the word and naming the ink color of the word, with the former disturbing the latter [43,44]. It is believed that individuals automatically read the word in order to grasp the meaning. If participants perceive that the stimulus even partly contains a word, the activation of the meaning of that word begins even if they attend to a different stimulus feature, e.g., color [43]. An alternative explanation is that words are associated with speaking the meaning, not its color, and therefore individuals have difficulty recruiting the necessary response to indicate the color of that word [27]. Finally, the Stroop effect is possibly a combination of conflicts on both stimulus and response level. Prior studies comparing adolescents (up to age 17) and adults (from 18 to 35 years of age) report lower accuracy in adolescents, indicating that interference control still develops throughout adolescence [26,45,46]. The results of our study suggest that on the behavioral level, interference control continues to develop into the early 20s.

The P2 component has been found in different cognitive tasks, such as selective attention [47], working memory [48] and feature detection [49]. In selective attention paradigms, P2 is presumably involved in protection against interference that comes from irrelevant stimuli [43]. Selective attention enables us to ignore irrelevant stimuli and orient our conscious awareness to attended relevant stimuli [50]. According to EEG data, enhanced positivity toward color between 150 and 250 ms post-stimulus at fronto-central electrodes reflects sustained attention to color [12,51]. This fronto-central distribution can be taken as evidence of a special role of the prefrontal cortex in selective attention regulation [52]. In the current study, both younger groups, Early 20s and Mid 20s, had higher incongruent and congruent P2 amplitudes compared to Early 30s. The presence of higher P2 amplitudes in Early 20s and Mid 20s might be the result of still-inadequate higher-order control processes, which are necessary for focusing attention on relevant stimuli in order to prevent distraction by the competing stimuli.

Early 20s had higher incongruent and congruent N2 compared to Mid 20s, and lower congruent P3 compared to both Mid 20s and Early 30s, while similar differences in amplitudes can be observed in the incongruent condition as well. During the classical Stroop task, fronto-central N2 has often been associated with conflict monitoring [47,53]. It has been shown that N2 is a result of a response conflict arising from competing task-relevant and task-irrelevant information. Larger N2 amplitudes imply that individuals attend more to task-irrelevant information [54]. Larson and Clayson [55] reported that increased N2 amplitude, as for Early 20s in our study, may be the result of inefficient recruitment of cognitive resources after high-conflict trials, which may lead to poorer performance on the task. The Stroop-related P3 is most often associated with evaluation of the conflict, and it usually decreases when task difficulty increases [40,56]. This means that Early 20s had more difficulty processing stimuli compared to Mid 20s and Early 30s. One study [57] compared three age groups—early adolescents, late adolescents and adults—to examine the maturation of three performance monitoring ERPs using a flanker task: the error-related negativity (ERN), error positivity (Pe) and the N2. They demonstrated that N2 still matures during late adolescence and affects the development of action monitoring processes [57]. Jongen and Jonkman [41] compared typically developing 6–7 year olds, 8–9 year olds and 10–12 year olds with adults aged 18–29 years using a standard color–word Stroop task and found differences in P3 and N450 amplitudes, indicating that children probably use more general brain activation to resolve the conflict, showing reduced ability to successfully apply interference control. Indeed, some fMRI studies suggest that adolescents use the same circuitry as adults, just less effectively [7,45,58]. More specifically, response inhibition relies on the interaction of the frontal control systems, including the dorsolateral and ventrolateral prefrontal cortex, with the basal ganglia and motor regions, along with the primary sensorimotor cortex, presupplementary and supplementary motor areas [5,11,24,45]. The present study fills the “age gap”, showing that the maturation of processes underlying effective action monitoring still occurs during the early 20s.

Finally, Early 20s also had higher incongruent N450 compared to Mid 20s and Early 30s. The N450 over the frontal–central regions is sensitive to the degree of interference between color and word information on incongruent Stroop trials [27]. As mentioned above, Spronk and Jonkman [26] found more errors and higher N450 in adolescents compared to adults, indicating poorer interference control in adolescence. The increased N450 in Early 20s in this study is possibly due to limited resource availability for top-down control. This is especially the case when top-down executive control is faced with high requirements such as suppressing Stroop-induced conflicts, which is in line with the protracted development of frontoparietal networks used for interference control in the early 20s [59,60]. Indeed, reduced accuracy in the Stroop task in Early 20s and Mid 20s compared to Early 30s was already suggested to be caused by the prolonged period of development of the frontal brain networks known to be involved in suppressing irrelevant stimuli [26].

Histological studies reported significant changes throughout adolescence in the prefrontal cortices [61,62], indicating continuation of the processes of synaptic proliferation and pruning into early adulthood. Neuroimaging studies provided further evidence of structural brain restructuration after adolescence, especially in the prefrontal cortices, all the way up to the mid or even late 20s [5,6,30]. Since prefrontal brain regions are responsible for higher cognitive functions, such as executive functions, continuing improvement of these functions can also be expected at this age. Therefore, in this study we used a narrow age range focusing on early adult years to investigate the behavioral and neurophysiological development of one of the core executive functions, interference control, after adolescence. Since intellectual abilities [63] and personality traits [64] can affect the performance on the executive function tasks as well as ERP amplitudes [65], all participants in this study were pretested with a battery of psychological tests to estimate non-verbal IQ, speed of information processing, neuroticism, extraversion, psychoticism and impulsiveness.

Everyday challenges during the early 20s are generally high and consist of different circumstances that require highly functioning cognitive skills. In our previous studies we investigated other components of executive functions, such as motor inhibition [8], performance monitoring [7,9] and cognitive control [10], using a large group of participants ranging from 19 to 44 years of age. Here, we applied a more complex task that calls for fully functioning interference control to provide a full picture of the development of various stages of one of the key components of executive functions—the inhibitory control—from motor inhibition to cognitive inhibition reflected in interference control across young adulthood. This series of studies that we conducted, investigating the development of inhibitory control across young adulthood, shows the importance of including narrow age ranges in developmental studies. Our findings challenge the existing models of cognitive development and suggest that future research should divide young adults into different age groups based on the findings from histological and neuroimaging studies. The theoretical framework should involve explanation on how young people develop into individuals who can effectively control their behavior and manipulate their skills in order to manage enormous everyday changes in roles and responsibilities related to career, family, friends and romantic partners—perhaps even by adding a new period of development from late adolescence through the early twenties, with a focus on ages 18–25, as proposed by Arnett [66]. Since the data in this study comes from a cross-sectional sample, future studies could benefit from a longitudinal design, which would help in reducing between-subject variation and establishing sequences of events. Other brain imaging methods, such as fMRI, could help in identifying brain regions implicated in developmental changes in interference control after adolescence.

## 5. Conclusions

We found evidence on the behavioral and neurophysiological levels that interference control is still not at adult level in the early 20s. If we consider results from cellular and structural studies, it seems that while synaptic pruning and myelination are ongoing, the cognitive performance is less efficient and is improving during the early 20s. Only later, after the myelination of all interconnections in the association cortex and pruning of excess synapses into specialized, efficient networks, is the improvement of cognitive functions achieved. Translated to the cognitive and behavioral levels, once the highest level of executive functioning is achieved, individuals can effectively control their thoughts and actions.

## Figures and Tables

**Table 1:** Summary of statistics for psychological testing battery.

Variable	Early 20s *M* (±*SD*)	Mid 20s *M* (±*SD*)	Early 30s *M* (±*SD*)	*F*_(2,128)_	*p*	ŋ_p_^2^
Non-verbal IQ	33.3 (±4.45)	32.1 (±5.24)	31.6 (±6.00)	1.2	0.30	0.02
Info. processing	45.0 (±5.24)	45.4 (±5.33)	43.8 (±4.99)	1.1	0.35	0.02
Impulsivity	29.6 (±4.52)	29.9 (±5.32)	30.1 (±4.88)	0.1	0.87	0.00
Psychoticism	3.8 (±2.34)	4.0 (±2.10)	4.3 (±2.09)	0.7	0.52	0.01
Extraversion	14.5 (±4.54)	15.0 (±4.43)	14.4 (±4.07)	0.2	0.83	0.00
Neuroticism	9.1 (±4.68)	8.2 (±5.53)	6.9 (±4.55)	2.1	0.12	0.03

**Table 2:** Summary of statistics for task performance.

Variable	*F*_(2,128)_	*p*	ŋ_p_^2^
*Accuracy*			
Incongruent	4.1	0.02 *	0.06
Congruent	1.8	0.17	0.03
*Reaction Time*			
Incongruent	0.9	0.42	0.01
Congruent	1.2	0.30	0.02

Note. * *p* < 0.05.

**Table 3:** Summary of statistics for ERPs.

Component	Condition	*F*_(2,128)_	*p*	ŋ_p_^2^
*Fz electrode*				
P2	Incongruent	**4.1**	0.02 *	**0.06**
	Congruent	2.9	0.06	0.04
N2	Incongruent	**4.9**	0.01 *	**0.07**
	Congruent	**3.8**	0.02 *	**0.06**
P3	Incongruent	2.8	0.06	0.04
	Congruent	**4.9**	0.01 *	**0.07**
N450	Incongruent	**6.1**	<0.01 **	**0.09**
	Congruent	2.4	0.10	0.04
*Cz electrode*				
P2	Incongruent	**5.4**	<0.01 **	**0.08**
	Congruent	**5.9**	<0.01 **	**0.08**
N2	Incongruent	2.7	0.07	0.04
	Congruent	2.6	0.07	0.04
P3	Incongruent	1.1	0.33	0.02
	Congruent	0.7	0.50	0.01
N450	Incongruent	2.9	0.06	0.04
	Congruent	2.0	0.14	0.03

Note. * *p* < 0.05; ** *p* < 0.01. Group differences are bolded.

## Data Availability

Research and analysis materials are available upon request. The data are not publicly available due to privacy.
